# Evaluation of Immunity for Mumps among Vaccinated Medical Students

**DOI:** 10.3390/vaccines9060599

**Published:** 2021-06-04

**Authors:** Cristiana Ferrari, Marco Trabucco Aurilio, Andrea Mazza, Antonio Pietroiusti, Andrea Magrini, Ottavia Balbi, Matteo Bolcato, Luca Coppeta

**Affiliations:** 1 Department of Occupational Medicine, University of Rome Tor Vergata, Viale Oxford 81, 00133 Roma, Italy; cristiana.ferrari@ptvonline.it (C.F.); andrea.mazza@ptvonline.it (A.M.); pietroiu@uniroma2.it (A.P.); andrea.magrini@uniroma2.it (A.M.); ottaventidue@hotmail.it (O.B.); lcoppeta@gmail.com (L.C.); 2 Department of Medicine and Health Sciences “Vincenzo Tiberio”, University of Molise, 86100 Campobasso, Italy; marco.trabuccoaurilio@unimol.it; 3 Legal Medicine, University of Padua, Via Falloppio 50, 35121 Padova, Italy

**Keywords:** mumps, outbreak, vaccination, health care workers, students

## Abstract

Healthcare workers are considered at higher risk for mumps infection than the general population. Since 2017, the national immunization plan recommended the administration of a dose of measles–mumps–rubella (MMR) vaccine to the healthcare operators who are unable to demonstrate a complete vaccination history or that are seronegative for at least one of the three agents. Regarding mumps infection, based on actual concerns regarding the loss of protection over the years after vaccination, the Advisory Committee on Immunization Practices (ACIP) recommended to administer a third dose of vaccine to operators previously vaccinated with two doses of MMR vaccine who belong to a group at increased risk of mumps infection in the event of an epidemic. This guideline, however, is not currently followed in Italy, resulting in a potential risk for vaccinated operators to become unprotected from mumps over the years. The aim of our study is to evaluate the persistence of a protective antibody level for mumps among medical students vaccinated during infancy or adolescence, at the start of their hospital internship. We retrospectively evaluated mumps-specific IgG levels in a group of medical students, in the period from 1 January to 31 December 2020. We evaluated the persistence of the detectable level of mumps-specific antibodies in relation to their vaccinal status, gender and time elapsed from vaccination. We found that 17.4% (65 subjects) of our sample were seronegative for mumps. The univariate analysis showed a significant difference in serological protection between male and female gender (77.0% vs. 86.2%; *p* < 0.05 with chi^2^ test) and between age classes (86.5% vs. 76.4%; *p* < 0.05 for subjects aged 18–23 years and over 23 years, respectively). Female gender was significantly related to higher serological protection even after adjusting for age classes and number of vaccine doses administered in a multivariate analysis model. Our study shows a substantial percentage of subjects lacking a protective mumps titer among medical students who were vaccinated in childhood. Given the higher risk of infection among those subjects, routine pre-employment screening should be performed among those operators regardless of their vaccination history and a third dose of MMR should be offered to unprotected students.

## 1. Introduction

Mumps is an infectious disease caused by a paramyxovirus, a member of the Rubulavirus family, characterized by puffy cheeks and tender swollen jaw due to parotid inflammation, but other common symptoms are fever, headache, myalgia, tiredness and loss of appetite [1,2].

In 2018, 11,312 cases of mumps were reported to ECDC by 28 EU/EEA Member States with an overall notification rate of 2.6 cases per 100.00 population. There were no deaths reported and hospitalization or complications due to mumps were rare, affecting 4.5% and 6.5%, respectively, of the cases with data recorded for these outcomes. In Italy during 2018, 777 cases of mumps were reported, while there were 829 cases in 2017 [3].

Mumps infection was more common among males than females across all age groups in the EU/EEA, with an overall male to female notification rate ratio of 1:3. Those aged 10–19 years experienced the highest age-specific notification rates and the highest proportion of cases vaccinated with two or more doses of the measles, mumps and rubella (MMR) vaccine.

Recent findings regarding the impact of the COVID-19 pandemic on the decreased childhood vaccination coverage raised questions about a significant increase in future mumps cases, irrespective of the possible decline in the transmission rate of the infection [4].

Despite evidence of incomplete protection or waning immunity following vaccination, high MMR vaccination coverage is considered crucial to prevent mumps outbreaks, reduce disease severity and achieve measles and rubella elimination goals [5].

Healthcare workers (HCWs) are considered to be at higher risk for mumps infection than the general population [6,7]. Healthcare-associated mumps is a public health concern because non-immune HCWs could infect susceptible patients and colleagues and, in a hospital setting, mumps disease can spread very quickly [6,7,8].

Compared to measles and rubella, the mumps vaccine is less immunogenetic so a significant proportion of HCWs could lack protective antibody titers at the time of employment, even after a complete two-dose MMR vaccination [9,10]. Although the protective cut-off level for mumps IgG remains uncertain, it is well known that subjects who show low antibody levels, if exposed to mumps virus, could become infected [11].

Since 2017, the Italian national immunization plan has recommended the administration of a dose of measles–mumps–rubella (MMR) vaccine to the healthcare operators who are unable to demonstrate a complete vaccination cycle of MMR or that are seronegative for at least one of the three agents [12].

Regarding mumps infection, based on actual concerns regarding the fall in protection over the years, the Advisory Committee on Immunization Practices (ACIP) recommended to administer a third dose of a vaccine for mumps for HCWs previously vaccinated with two doses of MMR vaccine who belong to a group at increased risk of mumps infection in the event of an epidemic [13,14]. This recommendation, however, is not actually followed in Italy where two doses of MMR is the standard vaccine schedule, resulting a potential risk for vaccinated operators to be unprotected from mumps.

The aim of our study is to evaluate the serological persistence of immunity to mumps, among medical students vaccinated during infancy or adolescence, at the start of their hospital internship.

## 2. Materials and Methods

We retrospectively evaluated mumps-specific IgG immunity status of all the medical students, vaccinated during infancy or adolescence, who underwent pre-employment screening for their internship at Foundation Polyclinic of Tor Vergata (PTV) at Rome in the period from 1 January to 31 December 2020. The study was approved by the local Ethical Committee.

We evaluated the persistence of a detectable level of mumps-specific antibodies in relation to their vaccinal status (including vaccine schedule), gender and age.

We included in the study all the students with a fully documented measles–mumps–rubella (MMR) vaccination history. Students who were unable to formally provide individual documentation were requested to obtain it from the local health authority. We evaluated the titer of mumps-specific IgG antibodies both in subjects who received one dose and in those with the two recommended doses (at a standard time elapsed since the first one of 28 days). Subjects with incomplete vaccination data or that were unable to provide any written documentation were excluded from the study.

The students were venipunctured for the purpose of venous blood sampling for the routine blood analysis and to detect the level of MMR-specific IgG antibodies. The test tubes were delivered to the laboratory for the analysis.

DiaSorin LIAISON^®^ Saluggia (VC) Italy Mumps IgG was used to detect the mumps-specific antibody titers; this is an indirect quantitative test that uses a chemiluminescence immunoassay (CLIA), the solid phase is a recombinant nucleoprotein expressed in P. pastoris and it uses MoAb to human IgG conjugated to isoluminol derivative technology. The sensitivity and specificity of the method are 98.5% (95% C.I.: 96.5–99.5%) and 98.2% (95% C.I.: 94.8–99.2%), respectively [14], and it needs 20 µL of serum or plasma.

According to the manufacturers’ instructions, we considered a mumps-specific IgG level higher than 11 AU/mL as protective.

Quantitative data were reported as mean ± SD, categorical variables were expressed as number (percentage and 95% confidence interval, 95% C.I.) of subjects. Univariate logistic regression models were used to calculate the association between serological protection and the main population characteristics (gender, age class, number of vaccine shots). Our multivariable analysis included as a dependent variable only factors that were statistically significant in univariate analysis. The results were considered statistically significant at a *p*-value level <0.05. Statistical analysis was performed using IBM SPSS software (Release 26).

## 3. Results

We enrolled in the study 373 medical students (148 males and 225 females); the median age of the population was 22.76 years (range: 18–26, DS: 1,82), which did not differ between genders.

Mean titer of protective mumps antibodies was 123.73 AU/mL (range 5.00–300.00, DS: 106.58). The main population characteristics are shown in Table 1. We found that 17.4% (65 subjects) of our sample was seronegative for mumps.

The univariate analysis showed was a significant difference in serological protection between male and female gender (77.0% vs. 86.2%; *p* < 0.05 chi^2^ test) and between age classes (86.5% vs. 76.4%; *p* < 0.05 for subjects aged 18–23 years and over 23 years, respectively). Female gender was significantly related to higher serological protection even after adjusting for age classes and number of vaccine doses administered in a multivariate analysis model with a *p*-value <0.01 (Table 2).

The median titer varied between genders; female subjects showed a mumps IgG titer of 128.35 U/mL (95% C.I. 114.38–142.32), while in the male group, the mean antibody level was 116.52 (95% C.I. 99.06–133.99), but the difference was not significant with an ANOVA test.

## 4. Discussion

The study was focused on the serological immunity for mumps in a population of medical students vaccinated with the MMR vaccine in childhood or adolescence, at the time of the beginning of their internship.

We found that a significant proportion (17.4%) of the subjects lacked serological protection for mumps, being seronegative or showing low antibody titers.

Since the WHO recommends an MMR vaccine coverage >95% to achieve herd immunity [6], our findings are worrisome and raise questions regarding the effectiveness of the current Italian immunization plan for mumps.

In a large multicenter study from Italy that investigated the vaccine coverage (VC) rate for most vaccine-preventable diseases among HCWs, inadequate VC was found for all vaccinations examined, and coverage was very low for measles, mumps, rubella, pertussis, chickenpox and influenza. Only 21.3% of subjects were vaccinated for mumps in the age class 20–30 years [15].

In a study on HCWs employed in northern Italy, the serological protection rate for mumps was 80% [16]. Similar rates of serological protection were found in recently published studies, in Italy and in other EU countries [17,18,19].

We found that most not non-immune subjects were in the age class of over 23 years, a group of subjects born before the year 1997, a period in which the vaccine coverage rate for mumps was low, despite the vaccine’s availability. Published studies reported a similar rate of MMR coverage and high risk of infection in young adults compared to children [20,21,22,23].

Unprotected operators were found mostly among male subjects; gender was the most predictive factor even after controlling for age and number of vaccine shots received by operators.

Gender differences in the response to vaccination are widely reported in the literature [24]. Women usually develop both a stronger immune response (considering antibody levels), and more severe adverse effects following vaccination; these findings can be attributed to enhanced activation of the immune system compared to male subjects [25]. Otherwise, clinical manifestations and the disease course of mumps infections are more severe among male subjects and complications are more frequent in those individuals. The results of our study highlight the need to improve preventive measures targeting male operators, since almost ¼ of them were unprotected at the serological screening.

Even if serology for EIA is the most frequently used method to evaluate the humoral response to mumps vaccine, the total IgG titer does not necessary correlate with immunity for the infection and disease, because the test measures both neutralizing and non-neutralizing IgG [26,27,28].

Our study shows that the mumps IgG titers decreased over the years and, even among subjects who received two doses of the MMR vaccination, the rate of unprotected subjects was alarmingly high.

Hospital transmission of mumps can occur and outbreaks are a rare but worrisome event, which may result in health and economic consequences [29].

The most effective strategy for controlling the spread of mumps during an outbreak is to identify the unprotected population and to vaccinate subjects lacking presumptive evidence of immunity or to exclude workers that cannot be vaccinated from settings with a higher risk of infection [30].

Recent findings in the literature highlighted that mumps outbreaks can occur in heavily vaccinated populations, such as HCWs, and mainly in young adults. Many factors have been considered to explain mumps outbreaks in those highly vaccinated populations: heterogeneous vaccine uptake and primary and secondary vaccine failure (immune escape and waning vaccine-induced immunity) [31].

Waning immunity and immune escape are the most reliable reasons of secondary vaccine failure. Waning vaccine-induced immunity accounts for the fall in vaccine immunity over time, determining an inadequate immune response, that leads to virus spread, making adults at higher risk to be infected than children. Potential waning immunity has been reported in recent mumps outbreaks that occurred in Europe that involved young adults who received two doses in childhood [30,31,32,33].

For the abovementioned reasons, in October 2017, the ACIP recommended to administer a third dose of mumps virus-containing vaccine to persons previously vaccinated with two doses who are at increased risk for acquiring the infection because of an outbreak. This recommendation is based on the fact that a two-dose cycle can be adequate for mumps control in the general population, but may not be able to prevent mumps outbreaks in prolonged, close-contact settings [13].

The national immunization plan in Italy stated to administer two doses of MMR vaccine to HCWs who are unable to demonstrate a complete two-dose vaccination history or who are unprotected at serological evaluation.

Italian regional health authorities coped with HCW vaccine hesitancy in order to improve vaccine coverage rate among those subjects: several Italian regions, such as Emilia Romagna and Puglia, enacted regional laws introducing mandatory vaccinations for non-immune HCWs [34,35]. Data from the literature show that mumps IgG titers decreased over time [27], both in people who received one dose and two doses of vaccine, so the vaccination history alone should be considered an unreliable indicator of immunity. Our study confirms those findings: due to the high rate of completely vaccinated but unprotected subjects, serological screening and administration of a booster dose should be considered at least in those at high risk of mumps infection.

Moreover, the workplace evaluation of serological status for mumps (as for measles and rubella [26]) in HCWs was shown to be highly cost-effective as it allows occupational medicine specialists to avoid the inappropriate administration of vaccine to subjects who were previously vaccinated (and also protected) but who are unable to provide written certification. Based on the results of previous studies, in HCWs, the evaluation of the mumps IgG level may be considered more accurate than direct immunization, allowing the correct identification of subjects’ status irrespective of a previous vaccination cycle (complete or incomplete).

## 5. Conclusions

Our study shows a substantial percentage of subjects lacking a protective mumps titer among medical students who were vaccinated in childhood. Given the higher risk of infection among those subjects, routine pre-employment screening should be performed among those operators regardless of their vaccination history and a third dose of MMR should be offered to unprotected students.

## Figures and Tables

**Table 1:** Main population characteristics.

		Total	N %	Mean Age (SD)	Mean Titer (SD)
Gender	Female	225	60.3%	22.76 ± 1.82	123.73 AU/mL ± 106.58
	Male	148	39.7%		
Age class	18–23	229	61.4%		
	>23	144	38.6%		
Vaccination	1 dose	87	25.0%		
	2 doses	261	75.0%		

**Table 2:** Main results.

Variables		Total	Immune	N%	95.0% C.I.	Univariate Logistic Regression Analysis	Multivariate Logistic Regression Analysis
Gender	Female	225	194	86.2%	81.0–90.4	*p* < 0.05	*p* < 0.01
	Male	148	114	77.0%	69.4–83.5		
Age class	18–23	229	198	86.5%	81.3–90.6	*p* < 0.05	
	>23	144	110	76.4%	68.6–83.1		
Vaccination	1 dose	87	72	82.8%	73.2–90. 0	n.s.	
	2 doses	261	225	86.2%	81.4–90.1		

n.s.: not statistically significant.

## Data Availability

The data presented in this study are available by request from the corresponding author. The data are not publicly available for ethical reasons.
